# The Interplay between Long Noncoding RNAs and Proteins of the Epigenetic Machinery in Ovarian Cancer

**DOI:** 10.3390/cancers12092701

**Published:** 2020-09-21

**Authors:** Naiade Calanca, Cecilie Abildgaard, Cláudia Aparecida Rainho, Silvia Regina Rogatto

**Affiliations:** 1 Department of Chemical and Biological Sciences, Institute of Biosciences, São Paulo State University (UNESP), Botucatu 18618-689, Brazil; naiade.calanca@unesp.br (N.C.); claudia.rainho@unesp.br (C.A.R.); 2 Department of Oncology, University Hospital of Southern Denmark-Vejle, Institute of Regional Health Research, University of Southern Denmark, 5000 Odense, Denmark; Cecilie.Abildgaard@rsyd.dk; 3 Department of Clinical Genetics, University Hospital of Southern Denmark-Vejle, Institute of Regional Health Research, University of Southern Denmark, 5000 Odense, Denmark

**Keywords:** lncRNAs, epigenetic regulators, transcriptional regulation, ovarian cancer, lncRNA databases

## Abstract

**Simple Summary:**

Epithelial ovarian cancer is an aggressive disease associated with relapse, resistance to chemotherapy, and high mortality rates. Recent discoveries have pointed out that long noncoding RNAs (lncRNAs) are potential biomarkers or therapeutic targets in several tumor types. However, fundamental knowledge about their functions and regulation is still lacking. Here, we present the current understanding of the interplay between lncRNAs and the epigenetic machinery influencing ovarian carcinogenesis. We also provide an overview of bioinformatics tools and databases that can be exploited for lncRNAs investigations. Altogether, this information can support the development of clinical initiatives to monitor disease progression or discover new strategies for the therapeutic management of ovarian cancer.

**Abstract:**

Comprehensive large-scale sequencing and bioinformatics analyses have uncovered a myriad of cancer-associated long noncoding RNAs (lncRNAs). Aberrant expression of lncRNAs is associated with epigenetic reprogramming during tumor development and progression, mainly due to their ability to interact with DNA, RNA, or proteins to regulate gene expression. LncRNAs participate in the control of gene expression patterns during development and cell differentiation and can be cell and cancer type specific. In this review, we described the potential of lncRNAs for clinical applications in ovarian cancer (OC). OC is a complex and heterogeneous disease characterized by relapse, chemoresistance, and high mortality rates. Despite advances in diagnosis and treatment, no significant improvements in long-term survival were observed in OC patients. A set of lncRNAs was associated with survival and response to therapy in this malignancy. We manually curated databases and used bioinformatics tools to identify lncRNAs implicated in the epigenetic regulation, along with examples of direct interactions between the lncRNAs and proteins of the epigenetic machinery in OC. The resources and mechanisms presented herein can improve the understanding of OC biology and provide the basis for further investigations regarding the selection of novel biomarkers and therapeutic targets.

## 1. Introduction

Despite the advances in diagnosis and treatment in the last decades, ovarian cancer (OC) is still considered a clinical challenge due to the absence of improvements in long-term survival [1,2]. Based on the Global Cancer Observatory (GLOBOCAN) estimates of cancer incidence and mortality, OC is a health problem worldwide, and the second cause of death among gynecological malignancies, after cervical cancer [3]. In 2018, it accounted for 295,414 new cases and 184,799 deaths globally [4]. This disease is considered complex, being characterized by heterogeneity at molecular, histological, and clinical levels [5]. OC is classified into different subtypes according to cell origin (epithelial, sex-cord stromal, germ cell, and mixed-cell type) and histological features [6]. Epithelial ovarian cancer (EOC) is the most common type of malignancy that affects ovaries (approximately 90%). Furthermore, multiple histological subtypes characterize the EOC, including serous, endometrioid, clear cell, mucinous, and malignant Brenner. Of these, high-grade serous ovarian carcinoma (HGSOC) is the most commonly diagnosed type and accounts for 70–80% of deaths from the whole spectrum of OC subtypes [7]. This cancer type is commonly associated with relapse and chemoresistance [8]. OC is frequently diagnosed at advanced stages, partly because these tumors often exhibit nonspecific symptoms at the first stages of the disease, and due to the absence of effective screening strategies for early detection [9,10]. The aggressive nature of the disease, commonly causing transcoelomic dissemination, adds to the poor prognosis of less than 30% 5-year survival for stage III and IV tumors [11,12]. Platinum-based chemotherapy is the primary treatment option, often combined with a taxane-based drug, namely paclitaxel. Several targeted therapies such as bevacizumab, an inhibitor of vascular endothelial growth factor (VEGF) or PARP (Poly-ADP-ribose-polymerase) inhibitors of proteins can be administered first-line or as a second-line treatment for the recurrent disease [13,14]. Although most patients initially respond well to chemotherapy, it is estimated that 80% of OC patients at advanced-stage will experience recurrence of their primary cancer [8].

The improvement in high-throughput technologies, such as tiling arrays and Next Generation Sequencing (NGS), as well as bioinformatics analyses have extended the boundaries of our knowledge about the noncoding sequences in the human genome. Once considered junk DNA, these sequences underwent a paradigm shift after the discovery that the human genome is pervasively transcribed. Although approximately 80% of the human genome is transcribed into RNAs, only a small fraction of them is associated with protein-coding exons [15,16]. This suggests that most DNA sequences can be transcribed but not to encode proteins, generating noncoding RNAs (ncRNAs). This information has altered our perception of this RNA class and led to increased attention to their functional roles as regulators of physiological programs during development and human diseases, including cancer **[17]**. These ncRNAs are involved in regulatory and structural functions in cells and are usually are classified into two main categories according to the molecule size: long and short noncoding RNAs. Long noncoding RNAs (lncRNAs) are a group of transcripts with more than 200 nucleotides in length and were initially defined by the apparent absence of protein-coding potential [18].

Through direct interactions with DNA, RNA, or proteins, lncRNAs exert regulatory roles over their target genes at transcriptional, post-transcriptional, and epigenetic levels in a broad range of biological processes, including coordination of allelic expression, cell cycle control, stem cell pluripotency maintenance, differentiation, and apoptosis [19,20]. Considering the relevance of these processes in health and disease and the great diversity of lncRNAs encoded by the human genome, it is not surprising that these transcripts have been linked to almost all hallmarks of cancer [21]. Coined by Hanahan and Weinberg, these hallmarks correspond to the traits acquired by cells that allow malignant transformation [22]. The regulation of gene expression mediated by lncRNAs may involve the epigenetic mechanisms by direct interactions with proteins of the epigenetic machinery, including histone modifications and chromatin remodeling [23]. The interplay between differentially expressed lncRNAs, such as *MALAT1* (metastasis-associated lung adenocarcinoma transcript 1) and *CDKN2B-AS1* (cyclin-dependent kinase inhibitor 2B antisense RNA 1), and histone-modifying or chromatin-remodeling complexes has been implicated in transcriptional regulation, enabling progression of different cancer types [24]. A set of the OC-related lncRNAs acts as oncogenes or tumor suppressors, contributing to cellular transformation [25,26,27].

The aberrant expression levels of these lncRNAs can be detected in tumor tissues and body fluids. Some lncRNAs were found to be widely dysregulated, such as *HOTAIR* (HOX transcript antisense RNA), whereas others are specifically altered in one or a few tumor types [28,29]. In EOC, it was demonstrated that hundreds of lncRNAs are differentially expressed compared to benign and normal control tissues [30]. Furthermore, some of them, either individually or as part of molecular signatures, exhibit a significant association with clinical outcomes, such as disease-free (DFS) and overall survival (OS) [31,32]. For instance, increased levels of the lncRNA *HOTAIR*, previously reported in EOC tissues, showed an association with a surrogate DNA methylation profile that predicts poor survival in patients treated with carboplatin [33]. Recently published reviews provide additional examples of differentially expressed lncRNAs associated with OC prognosis [31,34,35]. In light of the growing body of evidence, it can be understood why lncRNAs could serve not only as prognostic and predictive markers but also as highly specific therapeutic targets. However, to date, few lncRNAs have been recognized as key players in the resistance to therapy in OC.

Overall, most of the well-characterized lncRNAs are associated with the gene expression regulation, commonly at transcriptional rather than at the post-transcriptional level. Importantly, lncRNAs may contribute to tumor biology by directly interacting with proteins of the epigenetic machinery or chromatin-remodeling complexes. The interplay between lncRNAs and these proteins results in epigenetic silencing or activation of target loci in a *cis* or *trans*-regulatory manner by altering chromatin structure, histone marks, or DNA methylation patterns of proximal regulatory elements. In this review, we revisit the opportunities to explore lncRNAs associated with the disruption of epigenetic reprogramming in OC. We also provided a guide for databases and tools that could help select disease-related lncRNAs for future functional studies and potential clinical applications.

## 2. LncRNA Genes and Their Expression Patterns

Although lncRNAs were previously recognized as noncoding genes, recent evidence has suggested that many of them contain small open reading frames that are strongly associated with ribosomes, which results in the translation of small peptides [36]. Several of these small peptides, often comprising fewer than 100 amino acids, are biologically functional and even implicated in cancer-suppressive or cancer-promoting activities [37]. Alternatively, some studies have linked mRNAs to coding-independent regulatory functions, blurring the differences between coding and noncoding transcripts, and highlighting the emergence of a new class of bifunctional RNAs [38].

LncRNAs are widely expressed in human tissues and, while less abundant, they are more tissue-specific than protein-coding genes [39,40]. Based on the genomic positions relative to protein-coding genes, lncRNAs can be classified into intergenic or intragenic, and sense, antisense, or bidirectional lncRNAs [41]. They share the same transcriptional machinery as protein-coding genes and usually undergo similar co-transcriptional and post-transcriptional processing events: 5’-capping, splicing, and 3’-polyadenylation [42]. Thus, standard RNA-seq protocols allow the identification of most lncRNA sequences [43]. In the evolutionary context, lncRNA exons evolve at a higher rate than protein-coding exons and lncRNA homologs exhibit limited sequence similarity in comparison with protein-coding genes [43]. As for genome diversity, lncRNAs surpass their protein-coding counterparts: lncRNA genes amount to nearly 60,000, against approximately 20,000 protein-coding genes [15,39].

From this myriad of lncRNAs, only a small part was deeply studied, and some patterns of action were proposed to depict their functions and help to predict the activities of other transcripts yet to be characterized. Molecularly, lncRNAs act by four mechanisms: as signals, reflecting biological contexts that are crucial for gene regulation in space and time; as scaffolds, binding to several proteins simultaneously and allowing the assembly of multi-subunit complexes; as guides, recruiting regulatory complexes to target loci in *cis* or *trans* via RNA–protein and RNA–DNA interactions; as decoys, regulating the availability of other biomolecules through sequestration [44]. A single lncRNA frequently presents more than one mechanism of action. Most lncRNAs are exclusively localized in the nucleus, although some lncRNAs can occupy the chromatin and subnuclear domains or the cytoplasm. These localization patterns are possibly related to a particular lncRNA function [45]. Nuclear transcripts can perform their regulatory roles by interacting with epigenetic regulators and, consequently, affect DNA methylation, chromatin architecture, or histone marks deposition in *cis* and *trans* [46].

Besides the linear isoforms, lncRNA genes can generate transcripts with circular structures, called circRNAs [47]. The *CDKN2A/B* (cyclin-dependent kinase inhibitor 2A/B) locus, for instance, is known for its linear antisense ncRNA *CDKN2B-AS1* that interacts with the histone-modifying complexes Polycomb Repressive Complex 1 and 2 (PRC1 and PRC2, respectively) and mediates transcriptional regulation. This mechanism was implicated in epigenetic silencing of tumor suppressor genes in the carcinogenesis process [48]. Apart from its linear isoform, the *CDKN2B-AS1* gene can produce circular transcripts by alternative splicing. The expression of these molecules was associated with atherosclerotic vascular disease risk [49]. Importantly, numerous circRNAs were found to be overexpressed in the serum of OC patients, whereas others, such as circ_0051240, circ-HIPK3 (homeodomain interacting protein kinase 3), and circRNA-UBAP2 (ubiquitin associated protein 2), were implicated in the control of OC cells malignant behavior [50,51]. CircRNAs are more stable in cells than their linear counterparts due to their covalent closed-loop structure. The lack of 5’end and poly(A)-tail come as a result of the circRNA biogenesis process, usually by lariat-driven circularization or back splicing, and avoid degradation by ribonucleases [52]. RNA-seq datasets obtained with poly(A)-independent methods are required for circRNA detection. However, the library preparation procedures still lack standardization, since there are several approaches to enrich the circRNA content (e.g., poly(A) depletion, rRNA depletion, and RNase R treatment), which lead to variability in results [53].

Pseudogenes are generally derived from their parental genes that underwent insertions, deletions, or nucleotide substitutions and lost their ability to encode for functional proteins. Therefore, pseudogenic transcripts can exert regulatory roles modulating the expression and/or the function of their cognate genes or unrelated coding genes [54]. Additionally, a subgroup of pseudogene loci can express lncRNAs, besides producing endogenous small interfering RNAs (siRNAs) [54]. The *PTENP1* (phosphatase and tensin homolog pseudogene 1) pseudogene, for example, encodes sense and antisense lncRNA transcripts that act to modulate the expression of its parental gene, the tumor suppressor *PTEN* (phosphatase and tensin homolog). Taking into account the experimental evidence of its involvement in tumor development and progression, either regulating *PTEN* or other genes, *PTENP1* can be considered a tumor suppressor [55].

The dysregulated expression of lncRNAs in cancer and their prominent spatial and temporal specificity compared with protein-coding genes reinforces their appeal as potential biomarkers and therapeutic targets [56]. Similar to lncRNAs, circRNAs present tissue/developmental stage-specificity and aberrant expression in cancer [57]. The increased stability, accompanied by their abundance in exosomes and body fluids, make circRNAs even more attractive for clinical applications [57,58]. As for pseudogenes, their expression is generally restricted to tumor tissues or increased in tumors compared to normal tissues [59]. In the fifth section, we present some invaluable resources to evaluate the abnormal expression levels and other features displayed by these noncoding transcripts in normal and pathological conditions.

## 3. LncRNAs and Their Association with Ovarian Cancer

In EOC, certain lncRNAs were found to be differentially expressed compared with benign and normal tissues. For instance, Wang et al. (2016) reported the up- or downregulation of 663 lncRNAs. The expression of the antisense lncRNA *RP11-597D13.9* was positively correlated with its nearby coding gene, *FAM198B* (family with sequence similarity 198 member B, also known as Golgi associated kinase 1B or *GASK1B*), which potentially participates in EOC progression [30]. Based on a systematic analysis of lncRNA and mRNA expression profiles from The Cancer Genome Atlas (TCGA), a platinum resistance-specific lncRNA-mRNA network was identified in HGSOC [60]. A total of 35 lncRNAs and 270 mRNAs showed 124 significant lncRNA-mRNA co-expression relationships in this network. The study revealed that the lncRNAs involved in platinum resistance primarily regulate metabolic pathways and indicates the prognostic and therapeutic potential of lncRNAs in HGSOC [60]. Another study, including TCGA-HGSOC datasets, identified a 10-lncRNA prognostic signature that enabled to group the patients into low-, mid-, and high-risk groups, indicating significantly shorter OS and DFS for women in the last group [32].

LncRNA expression profiles of OC patients have been analyzed from GEO (Gene Expression Omnibus) datasets. These studies have used different bioinformatics approaches and included distinct datasets, with a little overlap between them. Song et al. (2018) reported a signature with seven lncRNAs (*RP11-126K1.6*, *ZBED3-AS1*, *RP11-439E19.10*, *RP11-348N5.7*, *RNF144A-AS1*, *GAS5*, and *F11-AS1*) [61], whereas Liu et al. (2017) identified a signature with eight lncRNAs (*ZFAS1*, *RP5-1061H20.5*, *RP11-489O18.1*, *RP11-16E12.1*, *LINC01514*, *TUG1*, *RP11-136I14.5*, and *CTD-2555A7.3*) [62], both able to predict the response of HGSOC patients to platinum-based chemotherapy [61,62]. A signature composed of seven different lncRNAs (*XR_948297*, *XR_947831*, *XR_938728*, *XR_938392*, *NR_103801*, *NR_073113*, and *NR_036503*) was significantly correlated with a poor response to treatment with paclitaxel in EOC patients [63]. Another signature comprising six lncRNAs (*RUNX1-IT1*, *MALAT1*, *H19*, *HOTAIRM1*, *LOC100190986,* and *AL132709.8*) was associated with recurrence in OC [64]. Overall, these studies revealed several lncRNAs linked to EOC via cancer-related functions and pathways, according to GO (Gene Ontology) and KEGG (Kyoto Encyclopedia of Genes and Genomes) enrichment analyses.

To date, a few lncRNAs commonly associated with cancer were also involved in OC outcomes. A set of abnormally expressed lncRNAs, such as *H19* (H19 imprinted maternally expressed transcript), *HOTAIR,* and *CDKN2B-AS1* was associated with patient survival [35]. The overexpression of the lncRNA *HOTAIR* in EOC tissues was positively correlated with the FIGO (International Federation of Gynecology and Obstetrics) stage, histological grade, lymph node metastasis, and inversely correlated with OS and DFS [65]. The knockdown of *HOTAIR* suppressed cell proliferation, reduced invasion, and restored cisplatin-sensitivity, specifically by enhancing chemotherapy-induced cytotoxicity and apoptosis in cisplatin-resistant OC cell lines [66]. Upregulated lncRNAs *CCAT2* (colon cancer-associated transcript 2), *LSINCT5* (long stress-induced noncoding transcript 5)*, CERNA2* (competing endogenous lncRNA 2 for microRNA let-7b), *PVT1* (Pvt1 oncogene), *UCA1* (urothelial cancer-associated 1), and *NEAT1* (nuclear paraspeckle assembly transcript 1) have also been implicated in cancer-promoting mechanisms in OC [67]. An additional example is *GAS5* (growth arrest-specific 5), which was found to be downregulated in OC and associated with poor prognosis [68]. Experimental assays inducing *GAS5* overexpression inhibited cell proliferation, migration, and invasion, besides promoting apoptosis [68].

The oncogenic and tumor suppressor actions of *HOTAIR* and *GAS5,* respectively, have been also described for other lncRNAs. *LEMD1-AS1* (LEMD1 antisense RNA 1), for instance, inhibits OC progression by regulating an axis including miR-183-5p and TP53 (tumor protein p53) and leading to decreased cell proliferation, migration, and invasion [69]. *MAGI2-AS3* (MAGI2 antisense RNA 3) downregulates MYC (MYC proto-oncogene, bHLH transcription factor) transcriptional activity via targeting the miR-525-5p/ MXD1 (MAX dimerization protein 1) axis [70]. OC cell growth and motility are repressed as a consequence of *MAGI2-AS3* induced overexpression. Conversely, the silencing of *DLX6-AS1* (distal-less homeobox 6 antisense RNA 1) impaired proliferation, migration, and invasion abilities of OC cells, besides inducing their apoptosis and repressing tumor growth in vivo [25]. Increased levels of *DLX6-AS1* were detected in OC cells and tissues [25]. The authors demonstrated that *DLX6-AS1* acts by sponging the miR-195-5p and upregulating its miRNA-target FHL2 (four and a half LIM domains 2). Likewise, *TMPO-AS1* (thymopoietin antisense RNA 1) knockdown significantly suppressed OC cell proliferation, migration, invasion, and angiogenesis, also inhibiting tumorigenesis and angiogenesis in vivo [26]. *TMPO-AS1* was found to be upregulated in OC cell lines and clinical samples and exerts its oncogenic role by binding to E2F6 (E2F transcription factor 6) and modulating its occupancy on the promoter region of *LCN2* (lipocalin 2), whose expression is activated. These examples highlight the potential of targeting lncRNAs for OC treatment.

In addition to the molecular signatures presented earlier, single lncRNAs have also been shown to affect response to therapy in OC, although the understanding of the underlying mechanisms is still in its infancy [31]. The well-known oncogenic lncRNAs *MALAT1*, *HOTAIR*, and *PANDAR* (promoter of CDKN1A antisense DNA damage-activated RNA) were associated with cisplatin resistance [71,72,73]. The silencing of *MALAT1* was demonstrated to enhance sensitivity to cisplatin due to the reduced downstream NOTCH1 (notch receptor 1) signaling, which led to the repression of the multidrug resistance gene *ABCC1* (ATP binding cassette subfamily C member 1) [72]. *HOTAIR* acts as a sponge for the miR-138-5p. The knockdown of *HOTAIR* was recently demonstrated to reverse cisplatin resistance due to the increased availability of the miR-138-5p repressing *EZH2* (enhancer of zeste 2 polycomb repressive complex 2 subunit) and *SIRT1* (sirtuin 1) [74]. Both *MALAT1* and *HOTAIR* have been suggested as potential therapeutic targets to restore platinum sensitivity. The overexpression of *PVT1* and *HOTAIR* were implicated in cisplatin resistance in cell line models by regulating apoptotic factors or activating the NF-κB (nuclear factor kappa B) pathway, respectively [75,76]. Intriguingly, increased levels of *PVT1* after carboplatin-docetaxel treatment induced TP53 and TIMP1 (tissue inhibitor of metalloproteases-peptidase inhibitor 1) proteins expression concomitantly to decreased cell proliferation [77]. Acting by an unknown mechanism, the lncRNA *UCA1* promotes the expression of kinase *SRPK1* (SRSF protein kinase 1), which was associated with increased proliferation and reduced apoptosis of OC cells, favoring the development of cisplatin resistance [78]. Interestingly, Adriaens et al. (2016) suggested that targeting the lncRNA *NEAT1* can sensitize OC to platinum-based chemotherapy since its isoform 2 is involved in genomic integrity preservation by modulating ATR-CHK1 (ATM and Rad3-related-checkpoint 1) signaling and potentially affects double-stranded break (DSB) repair [79].

## 4. LncRNAs Involved in Epigenetic Mechanisms in Ovarian Cancer

**Figure 1:**
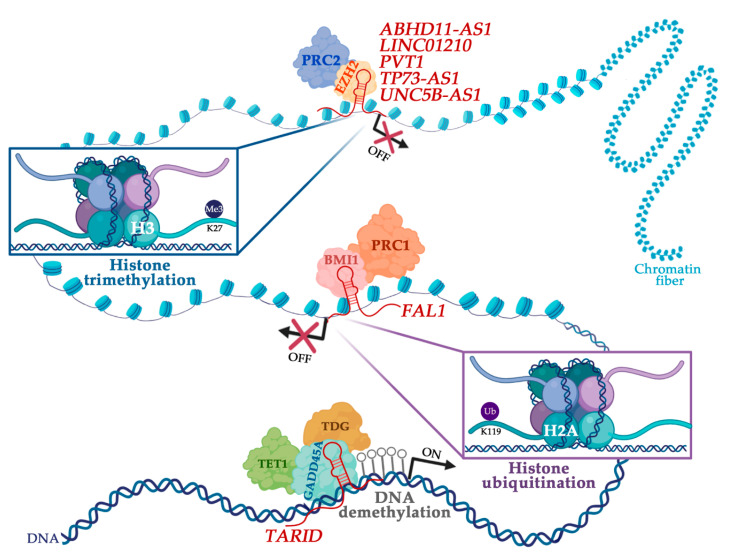
Long noncoding RNAs (lncRNAs) as epigenetic regulators in ovarian cancer. The representative lncRNAs implicated in each regulatory mechanism are shown in dark red. In the top and center, the interplay between lncRNAs and repressive histone-modifying complexes (Polycomb Repressive Complexes 2 and 1, respectively) are illustrated. The functional relationship between the lncRNA *TARID* (TCF21 antisense RNA inducing promoter demethylation) and the DNA demethylation is presented at the bottom of the figure. (Created with biorender.com).

The role of epigenetic machinery elements generally fits into one of three patterns of activity: (i) the writers can transfer chemical groups to nucleotide bases in DNA or amino acid residues in histone tails, (ii) the erasers are capable of removing these marks, and (iii) the readers are specialized in recognizing the DNA and histone epigenetic modifications. The writers include DNA methyltransferases (DNMTs), histone methyltransferases (HMTs), and histone acetyltransferases (HATs), while DNA demethylases, histone demethylases (HDMs), and histone deacetylases (HDACs) are their eraser counterparts. These molecules usually act in gene expression regulation by associating with lncRNAs and transcription factors [80,81]. In this section, we provide examples of lncRNAs whose interaction with proteins of the epigenetic machinery is supported by strong experimental evidence and potentially influence OC outcomes (Figure 1). These examples were obtained by searching either the lncRNA databases listed or from the published literature, i.e., referring to recent studies not included in databases (Table 1).

The knowledge of the interplay between lncRNAs, epigenetic machinery, and transcriptional regulation could significantly expand our understanding of how lncRNA dysregulation contributes to the epigenome and transcriptome alterations and, consequently, to tumor initiation and progression. LncRNAs participate in the epigenetic regulation by several mechanisms, including (i) interaction with DNMTs, guiding these proteins to a specific locus, leading to the promoter methylation and repression of tumor suppressor genes; (ii) by changing the nucleosome positioning and modifying chromatin accessibility to the transcriptional machinery; (iii) by recruiting histone-modifying enzymes in *cis* or *trans*. Another possible mechanism considers that lncRNAs can recruit demethylases and/or acetylases to the promoter region of oncogenes, and consequently activate them [82,83].

Hu et al. (2014) demonstrated the interaction between the lncRNA *FALEC* (focally amplified long noncoding RNA in epithelial cancer), also known as *FAL1*, and BMI1 (BMI1 proto-oncogene), a core component of PRC1. The authors reported the association of *FAL1* expression levels to the stabilization of BMI1 protein in human OC cell lines [84].

**Table 1:** Representative lncRNAs associated with disruption of epigenetic mechanisms in ovarian cancer.

LncRNA	Regulatory Mechanism	Target	Source	Refs.
*FAL1 (FALEC)*	Stabilization of BMI1 protein and PRC1-mediated deposition of repressive histone marks	*CDKN1A*	LncTarD and EVLncRNAs databases	[84,85]
*PVT1*	EZH2 recruitment	miR-214	LncTarD database	[86]
*TARID*	Recruitment of GADD45A and TET-dependent DNA demethylation	*TCF21*	LncTarD database	[87,88]
*LINC01210*	EZH2 recruitment and PRC2-mediated deposition of repressive histone marks	*KLF4*	Literature mining	[89]
*TP73-AS1*	EZH2 recruitment and PRC2-mediated deposition of repressive histone marks	*CDKN1A*	Literature mining	[90]
*UNC5B-AS1*	EZH2 recruitment and PRC2-mediated deposition of repressive histone marks	*NDRG2*	Literature mining	[91]
*ABHD11-AS1*	EZH2 recruitment and PRC2-mediated deposition of repressive histone marks	*TIMP2*	Literature mining	[92]

*ABHD11-AS1*: ABHD11 antisense RNA 1 (tail to tail); BMI1: BMI1 proto-oncogene, polycomb ring finger; *CDKN1A*: cyclin-dependent kinase inhibitor 1A; EZH2: enhancer of zeste 2 polycomb repressive complex 2 subunit; *FALEC*: focally amplified long noncoding RNA in epithelial cancer; GADD45A: growth arrest and DNA damage-inducible alpha; *KLF4*: Kruppel like factor 4; *LINC01210*: long intergenic nonprotein coding RNA 1210; *NDRG2*: NDRG family member 2; PRC1: Polycomb Repressive Complex 1; PRC2: Polycomb Repressive Complex 2; *PVT1*: Pvt1 oncogene; *TARID*: TCF21 antisense RNA inducing promoter demethylation; *TCF21*: transcription factor 21; TET: ten-eleven translocation family; *TIMP2*: TIMP metallopeptidase inhibitor 2; *TP73-AS1*: TP73 antisense RNA 1; *UNC5B-AS1*: UNC5B antisense RNA 1.

The PRC1 histone-modifying complex is known for binding the promoter regions of its target genes and mediating the deposition of one ubiquitin group to histone H2A at lysine 119 (H2AK119ub) [93]. However, the significance of this mark to the transcriptional repression of target genes is not yet fully understood. A novel mechanism was recently described for BMI1, which works in a PRC1-independent and non-epigenetic manner to regulate androgen receptor protein in castration-resistant prostate cancer [94]. Nevertheless, in Hu et al. (2014) study, the depletion of *FAL1* was correlated with the upregulation of *CDKN1A* (cyclin-dependent kinase inhibitor 1A). Experimental evidence suggests that the underlying mechanism involves, at least in part, the modulation of BMI1 occupancy and ubiquitination of H2AK119 on the promoter region of *CDKN1A*. The p21 protein encoded by *CDKN1A* promotes cell-cycle arrest and senescence. In vitro and in vivo studies demonstrated that both these processes were positively regulated by *FAL1* knockdown. Thus, *FAL1* has an oncogenic function in OC partly exerted by the epigenetic regulation of p21 expression levels, hinting at potential implications for cancer therapy [84].

The lncRNA *PVT1* recruits the protein EZH2 to the promoter of miR-214 [86]. EZH2 is one of two alternative catalytic subunits of PRC2 and is required, along with two other core components (SUZ12 polycomb repressive complex 2 subunit and embryonic ectoderm development), to produce the repressive histone modifications that characterize PRC2 activity: mono-, di-, and trimethylation of histone H3 at lysine 27. Under physiological conditions, the complex is recruited to unmethylated CpG islands and ensures the maintenance of previously established transcriptional silencing states, contributing to preserving cell identity [95]. In EOC, the upregulation of EZH2 has been reported by several studies and promotes proliferation, invasion, metastasis, and angiogenesis, besides its association with cisplatin resistance in OC cells [96].

In OC, both overexpression and downregulation of miR-214 have been described [97]. In the first case, this miRNA was implicated in reducing the cisplatin-induced apoptosis by targeting *PTEN*, whereas downregulation was linked to cancer cell stem-like properties and conversion of normal fibroblasts into cancer-associated fibroblasts [97]. Chen and colleagues identified increased *PVT1* and decreased miR-214 expression levels in EOC tissue samples and OC cell lines in comparison to normal tissue samples and human primary ovarian cells, respectively. In vitro results demonstrated the interaction between EZH2 and *PVT1* and the association of the lncRNA with proliferation, migration, and invasion [86].

Additional examples of lncRNAs capable of recruiting EZH2 to transcriptionally repress their target genes are *LINC01210* (long intergenic nonprotein coding RNA 1210), *TP73-AS1* (TP73 antisense RNA 1), *UNC5B-AS1* (UNC5B antisense RNA 1), and *ABHD11-AS1* (ABHD11 antisense RNA 1 (tail to tail)). Increased expression levels of *LINC01210* were reported in OC tissues, notably in metastatic or advanced stages of the disease, which were correlated with poor prognosis [89]. This lncRNA epigenetically downregulates the transcription factor KLF4 (Kruppel-like factor 4), favoring cell proliferation, invasion, and migration [89]. In OC, induced KLF4 overexpression promoted apoptosis and improved the efficacy of chemotherapy drugs (cisplatin and paclitaxel) [98]. Similar to the lncRNA *FAL1*, *TP73-AS1* mediates epigenetic repression of the tumor suppressor *CDKN1A*, although through with EZH2 instead of BMI1. *TP73-AS1* is upregulated in EOC tissues and cell lines, correlating with poor prognosis, and facilitating tumor progression by targeting *CDKN1A* [90].

The lncRNA *UNC5B-AS1* and its target gene *NDRG2* (NDRG family member 2) have been poorly explored in OC. *NDRG2* has been associated with tumor suppression in several cancer types and was found to be downregulated in OC tissues and cell lines [91]. In this context, *UNC5B-AS1* recruits EZH2 to inhibit *NDRG2* transcription, promotes cell proliferation, and impairs apoptosis [91]. To confirm bioinformatic predictions, *ABHD11-AS1* was experimentally proven to interact with EZH2 leading to the epigenetic repression of *TIMP2* (TIMP metallopeptidase inhibitor 2) and further promoting invasion and metastasis [92]. This evidence sheds light on the function of *ABHD11-AS1* in OC, albeit the inverse correlation between *EZH2* and *TIMP2* expression levels had already been detected in EOC tissues [99]. *TIMP2* is a member of the TIMP gene family, which encodes proteins that inhibit the activity of matrix metalloproteinases. The metalloproteinases degrade the extracellular matrix and facilitate metastasis initiation. Unsurprisingly, *TIMP2* aberrant expression promotes cell growth and invasion in several malignancies [92].

The antisense lncRNA *TARID* (TCF21 antisense RNA inducing promoter demethylation) mediates demethylation of the protein-coding gene *TCF21* (transcription factor 21), a tumor suppressor that is transcribed in the sense orientation. In OC samples, the CpG islands associated with the *TCF21* and *TARID* promoter regions are hypermethylated, and both genes are repressed. In contrast, in normal ovarian epithelium, the promoters are unmethylated, and both transcripts are expressed. It was shown that *TARID* binds to the promoter of its target gene and recruits GADD45A (growth arrest and DNA damage-inducible alpha), a stress response protein [88]. *GADD45A* expression level is commonly decreased in different cancer types. Interestingly, one functional single nucleotide polymorphism mapped to this gene is related to significantly decreased *GADD45A* mRNA levels and was implicated in OC susceptibility and prognosis [100]. Recent evidence has suggested that GADD45A recognizes the DNA-lncRNA hybrid formed at the CpG island of the *TCF21* promoter, rather than its sequence, and attracts the DNA demethylation machinery to activate the transcription of this gene. Consequently, GADD45A was proposed to be an epigenetic reader [101]. It recruits TDG (thymine DNA glycosylase), a component of the deamination-base excision repair machinery (BER), and proteins of the TET (ten-eleven translocation) family to the *TCF21* promoter [88]. TETs are epigenetic erasers known for their role as DNA demethylases in mammals; they oxidize 5-methylcytosine (5mC) to 5-hydroxymethylcytosine (5hmC), and afterward to 5-formylcytosine (5fC) or 5-carboxylcytosine (5caC). The residues of 5fC and 5caC can be replaced by unmodified cytosine following excision by TDG and repair of the abasic site by BER. This process is called active modification-active removal and occurs independently of DNA replication [102].

The examples of epigenetic mechanisms mediated by lncRNAs presented here could help to uncover new regulatory interactions between lncRNAs and epigenetic machinery components. Considering the PRC2 promiscuous binding to RNAs along with the estimate that more than 20% of human lncRNAs are associated with PRCs and chromatin-remodeling complexes, the recruitment of EZH2 and other less-studied mechanisms described herein are likely to apply to some of the still uncharacterized lncRNAs as well [103,104]. Furthermore, our portrayal of lncRNAs associated with epigenetic machinery elements provide frameworks that could be explored in future studies to characterize the function of OC-associated lncRNAs.

## 5. A Concise Guide to Exploring lncRNA Databases

The significant amount of data generated using low- and high-throughput methods for the identification and functional characterization of lncRNAs has been organized and updated in many user-friendly databases. Overall, these comprehensive databases provide basic information such as lncRNA annotation, lncRNA–target interactions, the affected biological processes, and lncRNA-mediated regulatory mechanisms in human diseases (transcriptional regulation, epigenetic regulation, chromatin looping, competing endogenous RNA or miRNA sponge, relationships with mRNAs and proteins). They are useful for analyzing and defining the best candidates for further investigations when considering the involvement of lncRNAs in human diseases. The databases summarized herein are publicly available and have information collected on the association between lncRNAs and human diseases. Thus, they are invaluable resources to discover novel biomarkers and potential therapeutic targets for ovarian tumors.

Concurrently, the selected databases are focused on different aspects of lncRNA biology. Therefore, we grouped them into three major categories that can be used as a guide to browse these resources. Based on their main focuses, i.e., annotation of lncRNAs, regulatory functions, or association with human diseases and phenotypes, we summarized this set of representative databases in Table 2. Table S1 includes features of these databases, such as data sources, coverage, and other relevant information. For instance, NONCODE, LNCipedia, LncRNAWiki, EVLncRNAs, LncRBase, and LncBook are mainly dedicated to annotating lncRNAs [105,106,107,108,109,110]. Although the data sources of these databases partly overlap, some of them also integrate information on lncRNAs from different species, their functions, sequence conservation, and other details. Most importantly, all of them include associations between lncRNAs and human cancers. Among these resources, LncBook is the most comprehensive in terms of the number of human lncRNAs, which amounts to 268,848 transcripts and 140,356 genes. Considering the disease-related lncRNAs gathered by this knowledgebase, *HOTAIR*, *MALAT1*, *H19*, *MEG3* (maternally expressed 3), *CDKN2B-AS1*, *PVT1*, *NEAT1*, and *GAS5* are some of the most studied. Each of these lncRNAs has been associated with at least 30 diseases, including OC [108].

The regulatory functions of lncRNAs could be especially explored in databases such as ENCORI, LncRNA2Target, LncTarD, and NPInter, which dive into interactions between these transcripts and different types of biomolecules [114,116,117,118]. Two other databases, LncACTdb and miRSponge, curate miRNA–sponge relationships [119,120]. Endogenous transcripts that share the same miRNA-binding sites can compete for the corresponding miRNA and regulate each other post-transcriptionally by titrating miRNA availability [135]. These transcripts, called competing endogenous RNA (ceRNAs), are comprised of noncoding and protein-coding RNAs, and the role of these regulators in cancer can be found in Salmena et al. (2011) and Yang et al. (2016) [136,137]. ENCORI provides a comprehensive collection of RNA–RNA and protein–RNA interaction networks. Lnc2Target collects lncRNA–target associations validated by high- and low-throughput methods. Emphasizing the contribution of lncRNAs to pathogenesis in humans, LncTarD only collects disease-related lncRNA–target regulations. On the other hand, NPInter curates functional interactions between ncRNAs and DNA, proteins, or other types of RNA across numerous organisms. Lately, the concern about offering tools that facilitate the analyses of the information available in databases has been raised, and each of these resources, except for LncRNA2Target and miRSponge, contains applications that illustrate the interactions between lncRNAs and their putative partners. Co-LncRNA and Lnc2Meth are also devoted to the elucidation of regulatory roles of human lncRNAs, the first analyzing downstream effects, and the second focusing on a mechanism [121,122]. Co-LncRNA employs RNA-seq data to assess the effects of individual or multiple lncRNAs in GO annotations and KEGG pathways, whereas Lnc2Meth links lncRNAs to DNA methylation status in several diseases.

Other databases were specially built to provide information on lncRNA–disease associations, such as LncRNADisease, Lnc2Cancer, MNDR, ncRPheno, and Lnc2Catlas [123,125,127,128,129]. LncRNADisease combines experimental and bioinformatic evidence to provide information on relationships between lncRNAs or circRNAs and diseases. It is the first lncRNA–disease association database that documents causal relationships, mostly cancer-related, which were identified based on strong experimental evidence from in vitro and/or in vivo models [124]. Lnc2Cancer strictly gathers associations supported by experimental evidence and human cancer subtypes, whereas MNDR is dedicated to both experimentally validated and predicted ncRNA–disease associations across six mammalian species. ncRPheno provides several web applications to facilitate the analysis and visualization of ncRNA–disease associations. Lnc2Catlas relies on three scoring methods to collect associations between lncRNAs and cancer. Additionally, some of the resources exemplified by CSCD, Circ2Disease, CircR2Disease, and MiOncoCirc cover the involvement of circRNAs in diseases exclusively [130,131,132,134]. MiOncoCirc is the first circRNA database to include datasets derived from clinical tumor samples. All MiOncoCirc samples were processed with the poly(A)-independent exome capture RNA-seq protocol, an approach that was proven to be more reliable than the often-used RNase R and rRNA removal methods. This database supports the identification of tissue-specific transcripts that could be further explored as biomarkers in cancer [134].

Bioinformatic tools also help to unveil the association between lncRNAs and different cancer types. The Atlas of Noncoding RNAs in Cancer (TANRIC) data portal is a platform for interactive analysis of clinical and genomic data coupled to lncRNA expression in cancer, including RNA-seq data from TCGA, independent studies, and cell lines from Cancer Cell Line Encyclopedia [138]. OncoLnc is an online resource that integrates survival data from TCGA and RNA-seq expression data for mRNAs, miRNAs, and lncRNAs to enable the exploration of survival correlations, besides allowing the download of the data used in the analyses [139]. Finally, LncDisease is a standalone software that relies on a sequence-based method to predict associations between lncRNAs and human diseases. The analysis involves two steps: (i) the prediction of miRNAs that interact with the inputted lncRNAs and (ii) the disease enrichment analysis of the predicted miRNAs, providing a list of significant diseases potentially associated with lncRNAs [140].

## 6. Conclusions and Perspectives

The altered expression profiles of lncRNAs in cancer, their tissue specificity, and regulatory roles highlight the importance of elucidating lncRNAs functions for new advances in OC diagnosis, prognosis, and therapy. Despite the promising findings, greatly accelerated by high-throughput and in silico approaches, and the large number of lncRNA in the human genome, few cancer-related lncRNAs have been characterized, annotated, and explored to shed light on their potential for clinical applications.

Currently, the development of epi-drugs, comprising compounds targeting enzymes involved in epigenetic regulations, has been limited by the lack of biomarkers for proper patient stratification for clinical trials [141]. Furthermore, interfering with a single epigenetic alteration can lead to global and local effects on chromatin conformation affecting several biological processes and pathways [142]. The regulation of the epigenetic machinery by multiple lncRNAs includes an additional layer of complexity. These transcripts can serve a dual purpose: allowing specific interference with selected targets and a more robust evaluation of patients’ responses to the treatment with epi-drugs. The technology for silencing specific lncRNA already exists; however, our knowledge of the widespread functions and interactions of lncRNAs is still limited.

In order to target lncRNAs for further investigations and the development of novel therapeutic strategies, ASOs (antisense nucleotides), siRNAs, and the CRISPR (clustered regularly interspaced short palindromic repeats) system are some of the available methodologies [21]. Each of these approaches offer different routes to achieve the lncRNA modulation and particular advantages and pitfalls that should be appreciated according to the features of the intended targets. ASOs, for instance, are single-stranded deoxyribonucleotide molecules that can hybridize with complementary lncRNAs and attract RNase H to degrade the target RNA. These oligonucleotides can be modified to improve their intracellular stability and are equally successful in silencing nuclear and cytoplasmic transcripts [143]. In contrast, siRNAs are less effective for the depletion of nuclear lncRNAs and, consequently, are preferable for cytoplasmic applications [144]. The CRISPR system can be used to knockout the target lncRNA genes when coupled with endonuclease Cas9, which produces DSBs in specific DNA sequences, or to repress the target lncRNA transcription when coupled with catalytically dead Cas9 (dCas9) fused to KRAB (Kruppel-associated box) chromatin modifier domain [143,145]. Hopefully, the information and guidelines provided here can help with the design of future studies employing the aforementioned methods to gain more insight into how lncRNAs can be exploited as biomarkers and therapeutic targets in OC.

## Figures and Tables

**Table 2:** Summary of lncRNA databases (last accessed on July, 2020).

Database	Aim	Website	Refs.
lncRNA annotation			
NONCODE v5.0	Collects and annotates a comprehensive set of ncRNAs, especially lncRNAs.	http://www.noncode.org/	[105]
LNCipedia 5.2	Collects sequences and annotation data for a comprehensive set of lncRNA transcripts.	https://lncipedia.org	[107,111]
LncRNAWiki	Collects and integrates comprehensive information on human lncRNAs relying on collaborative curation.	http://lncrna.big.ac.cn	[110,112]
EVLncRNAs	Collects lncRNAs that were validated by low-throughput experiments.	http://biophy.dzu.edu.cn/EVLncRNAs	[109]
LncRBase	Provides information on a comprehensive set of lncRNAs, ranging from basic transcript features to additional details on their genomic context.	http://bicresources.jcbose.ac.in/zhumur/lncrbase	[106]
LncBook	Collects a comprehensive set of human lncRNAs and provides multi-omics data integration, functional annotation, and disease association for these transcripts.	http://bigd.big.ac.cn/lncbook	[108,113]
**lncRNA regulatory functions (interactions, mechanisms, and effects)**			
ENCORI	Integrates multi-dimensional sequencing data to provide a comprehensive collection of RNA–RNA and protein–RNA interaction networks.	http://starbase.sysu.edu.cn/	[114,115]
LncRNA2Target v2.0	Collects lncRNA–target associations supported by high- and low-throughput methods.	http://123.59.132.21/lncrna2target/	[116]
LncTarD	Collects and illustrates experimentally supported lncRNA–target regulations in human diseases.	http://bio-bigdata.hrbmu.edu.cn/LncTarD	[117]
NPInter v4.0	Integrates literature reported evidence and high-throughput data processing to collect and illustrate functional interactions between ncRNAs and biomolecules.	http://bigdata.ibp.ac.cn/npinter	[118]
LncACTdb 2.0	Collects and illustrates information on ceRNA relationships between different types of RNAs in diverse diseases.	http://www.bio-bigdata.net/LncACTdb/	[119]
miRSponge	Collects experimentally validated miRNA sponges and ceRNA interactions.	http://bio-bigdata.hrbmu.edu.cn/miRSponge/	[120]
Co-LncRNA	Allows the identification of lncRNA coexpressed protein-coding genes and the assessment of the effects of individual or multiple lncRNAs in GO annotations and KEGG pathways.	http://bio-bigdata.hrbmu.edu.cn/Co-LncRNA/	[121]
Lnc2Meth	Integrates experimental and computational evidence to provide information on regulatory relationships between lncRNAs and DNA methylation in diverse human diseases.	http://bio-bigdata.hrbmu.edu.cn/Lnc2Meth/	[122]
**lncRNA–disease associations**			
LncRNADisease 2.0	Integrates experimental and computational evidence to provide information on lncRNA/circRNA–disease associations, including causal relationships.	http://www.rnanut.net/lncrnadisease/	[123,124]
Lnc2Cancer v3.0	Collects and integrates experimentally supported associations between lncRNAs and cancer subtypes.	http://www.bio-bigdata.net/lnc2cancer/	[125,126]
MNDR v2.0	Collects and integrates experimentally supported and predicted ncRNA–disease associations in mammals.	http://www.rna-society.org/mndr/	[127]
ncRPheno	Collects comprehensive ncRNA–disease association data and offers web applications to facilitate analysis and visualization of this data.	http://lilab2.sysu.edu.cn/ncrpheno	[128]
Lnc2Catlas	Integrates multiple methods and data sources to collect quantitative associations between lncRNAs and cancer.	http://lnc2catlas.bioinfotech.org/	[129]
CSCD	Collects and integrates information on cancer-specific circRNAs.	http://gb.whu.edu.cn/CSCD	[130]
Circ2Disease	Collects information on experimentally supported circRNAs in human diseases.	http://bioinformatics.zju.edu.cn/Circ2Disease/index.html	[131]
CircR2Disease	Collects information on experimentally validated associations between circRNAs and diseases.	http://bioinfo.snnu.edu.cn/CircR2Disease/	[132,133]
MiOncoCirc	Collects data on circRNA formation and abundance in cancer.	https://mioncocirc.github.io/	[134]

## Supplementary Materials

The following are available online at https://www.mdpi.com/2072-6694/12/9/2701/s1: Table S1: Publicly available lncRNA databases and their main features (last accessed on July 2020).
