# Supplementary material for: The Interplay between Long Noncoding RNAs and Proteins of the Epigenetic Machinery in Ovarian Cancer
Source: Cancers (Basel). 2020 Sep 21;12(9):2701. doi: 10.3390/cancers12092701 (PMC7563210; doi:10.3390/cancers12092701)
Supplement: Supplementary file 1 [file cancers-12-02701-s001.zip › cancers-922703-supplementary.docx]

**Supplementary Materials:**

The Interplay between Long Noncoding RNAs and Proteins of the Epigenetic Machinery in Ovarian Cancer

Naiade Calanca, Cecilie Abildgaard, Claudia Aparecida Rainho and Silvia Regina Rogatto

**Table S1.** Publicly available lncRNA databases and their main features (last accessed on July 2020).

| **Database** | **Data sources** | **Coverage** | **Main features** | **Website** | **Refs.** |  |
| --- | --- | --- | --- | --- | --- | --- |
| **lncRNA annotation** | | | | | |  |
| NONCODE v5.0 | Previous versions of NONCODE, relevant published literature from PubMed and annotation data from Ensembl, RefSeq, lncRNAdb, and LNCipedia; lncRNA-disease relationships from LncRNADisease, Lnc2Cancer, MNDR, and LncRNAWiki; exosome RNA-seq datasets from GEO; lncRNA-SNP associations from LincSNP. | All types of ncRNAs (except rRNAs and tRNAs) across 17 species, including 172,216 lncRNA transcripts and 96,308 lncRNA genes from humans. | Extensive annotation covering sequence, structure, expression, function, conservation, experimentally supported disease associations and many other aspects of lncRNAs, besides providing predicted lncRNA-SNP associations and expression profiles of lncRNAs in exosomes for human species. | http://www.noncode.org/ | [105] | |
| LNCipedia 5.2 | Annotation data from FANTOM CAT, Ensembl, LncRNAdb, Human BodyMap lincRNAs, NONCODE, GENCODE, RefSeq, and datasets of high-throughput analyses; relevant published literature from PubMed. | Exclusively human lncRNAs, including 127,802 transcripts and 56,946 genes. | Regularly updated annotation information on human lncRNAs, as well as protein-coding potential and locus conservation assessment, ORF prediction, and insights into functions of these transcripts through linked supporting papers. | https://lncipedia.org | [107,111] | |
| LncRNAWiki | Annotation data from GENCODE, NONCODE, and LNCipedia; information curated and updated by the scientific community. | 106,063 human lncRNAs grouped into seven categories according to their genomic location. | Basic lncRNA annotation data integrated with user-edited sections, including information on function, expression, regulation, associated diseases, conservation, and laboratories working on that transcript globally. | http://lncrna.big.ac.cn | [110,112] | |
| EVLncRNAs | Published literature curated from PubMed containing lncRNAs confirmed by low-throughput methods; annotation data from NCBI and Ensembl; functional and disease-related information from lncRNAdb, LncRNADisease, Lnc2Cancer and PLNlncRbase. | 1,543 experimentally validated lncRNAs across 77 species, including 869 human lncRNAs. | Basic lncRNA annotation data, as well as information on corresponding functions, interaction partners and associated diseases, if known; links to lncRNA prediction tools (CPC and COME). | http://biophy.dzu.edu.cn/EVLncRNAs | [109] | |
| LncRBase | Relevant published literature from PubMed; transcript annotation data from Ensembl, UCSC Genome Browser, NONCODE, Human BodyMap lincRNAs and H-InvDB; miRNA sequences from mirBase; piRNA sequences from NCBI; Imprinted genes and their annotations from Geneimprint; RNA-seq datasets from GEO; Affymetrix GeneChip Human Genome U133 Plus 2.0 Array and Affymetrix GeneChip Mouse Genome 430 2.0 Array probe sequences from manufacturer’s website; consensus gene expression signatures of different tumor tissues from Gene Expression Barcode. | Human and mouse lncRNA transcripts, grouped into 14 categories according to their genomic location, including 133,361 human lncRNA entries. | Basic lncRNA annotation data integrated with genomic location, overlapping small ncRNAs, and lncRNA-associated Repeat Elements, imprinted genes, and CpG islands; expression patterns in different tissues and information on association with disease. | http://bicresources.jcbose.ac.in/zhumur/lncrbase | [106] | |
| LncBook | LncRNA annotation data from GENCODE, NONCODE, LNCipedia and MiTranscriptome; RNA-seq datasets from HPA and GTEx; literature reported lncRNAs from LncRNAWiki; Bisulfite-seq data from TCGA and ENCODE; predicted lncRNA-miRNA interactions from TargetScan and miRanda, and experimentally validated interactions from starBase; genome variation data from dbSNP, 1000 Genomes Project, ClinVar and COSMIC; lncRNA-disease associations from LncRNADisease and LncRNAWiki | Exclusively human lncRNAs, including 268,848 transcripts and 140,356 genes. | Basic lncRNA annotation data, as well as information on corresponding expression and methylation patterns, genome variation, interaction with miRNAs, functions, and associated diseases; expert-curated resource that is complementary to community-curated LncRNAWiki. | http://bigd.big.ac.cn/lncbook | [108,113] | |
| **lncRNA regulatory functions (interactions, mechanisms, and effects)** | | | | | | |
| ENCORI | CLIP-seq datasets from GEO and published literature; annotation data from miRBase, GENCODE, Ensembl, and circBase; predicted miRNA target sites from TargetScan, miRanda, PITA, PicTar, and RNA22; functional annotation data from KEGG database, PANTHER database, MSigDB, and NCBI FTP site. | More than 1.1 million miRNA-ncRNA (small ncRNAs, lncRNAs, circRNAs, and pseudogenes), 2.5 million miRNA-mRNA, 2.1 million RBP-RNA, and 1.5 million RNA-RNA interactions across 23 species, including humans. | Prediction of RNA-RNA and protein-RNA interactions and ceRNA regulatory networks under user-selected stringency criteria in normal tissues and cancer cells, as well as graphical visualization of this information. | http://starbase.sysu.edu.c/ | [114,115] | |
| LncRNA2Target v2.0 | Relevant published literature from PubMed; annotation data from GENCODE, Ensembl, and NCBI GenBank; datasets of high-throughput analyses from GEO. | LncRNA-target associations in human and mouse, including 1,465 relationships from low-throughput experiments and 72,102 relationships from high-throughput experiments for humans. | Information on target genes differentially expressed after knocking down or overexpressing a lncRNA, or validated by binding experiments. | http://123.59.132.21/lncrna2target/ | [116] | |
| LncTarD | Published literature curated from PubMed containing functional regulations validated by strong experimental evidence (e. g. RNA pull-down assay, RNAi, qRT-PCR, and luciferase reporter assay); annotation data from Ensembl; RNA-seq datasets from TCGA; GENCODE reference transcriptome. | 2,822 lncRNA-target regulations in 177 human diseases, including 475 lncRNAs. | Annotation of the lncRNA-target regulations, including regulatory mechanisms and affected biological functions in human diseases; association with drug response and data on differential expression of the regulator and the target gene in several cancer subtypes; graphical overview of all this information. | http://bio-bigdata.hrbmu.edu.cn/LncTarD | [117] | |
| NPInter v4.0 | Relevant published literature from PubMed and previous version of NPInter; interaction data from RISE database; CLIP-seq and ChIRP-seq datasets from GEO, and eCLIP datasets from ENCODE; miRNA binding sites from miRanda and TargetScan; genome binding sites from GENCODE; annotation data from NONCODE, miRBase, circBase, UniProt, Ensembl, UniGene, and RefSeq; disease-related databases, including LncRNADisease, MNDR, eDGAR and circRNADisease; risk GWAS sites for RNA-DNA interactions from the GWAS Catalog. | RNA–RNA, RNA–protein, RNA-DNA, and RNA-transcription factor interactions covering most kinds of ncRNAs (except tRNAs and rRNAs) across 35 organisms, including 658,171 lncRNA interactions and 335 circRNA interactions. | Detailed annotation of interactions and each participating molecule; including related diseases, graphical visualization of ncRNA genome binding sites, and network viewing of interactions; identification of interactions involved in diverse biological processes using gene/lncRNA lists associated to certain functions (e.g. oncogenes from ONGene and alternative splicing factors from MiasDB). | http://bigdata.ibp.ac.cn/npinter | [118] | |
| LncACTdb 2.0 | Published literature curated from PubMed containing ceRNA associations supported by high confidence experiments (e. g. PCR, western blot, and luciferase reporter assays); AGO-CLIP-seq datasets from starBase; human miRNAs and their targets from TarBase and mirTarBase; predicted miRNA-lncRNA interactions from TargetScan, PITA, miRanda, and RNAhybrid; pathway annotation data and gene sets of cancer hallmark processes from MSigDB; Clinical follow-up information from TCGA. | 47,673 predicted ceRNA interactions in humans as well as 2,663 experimentally supported ceRNA interactions, including lncRNAs, mRNAs, circRNAs, and pseudogenes, across 23 species and 213 diseases/phenotypes. | Identification and visualization of experimentally supported and predicted ceRNA interactions from internal datasets or user-supplied expression profiles; genomic information on lncRNA transcripts displayed by a genome browser tool; functional annotation of ceRNAs based on different vocabularies; graphical visualization of survival, network and cancer hallmark information for predicted ceRNAs. | http://www.bio-bigdata.net/LncACTdb/ | [119] | |
| miRSponge | Published literature curated from PubMed containing miRNA-sponge and ceRNA interactions supported by high confidence experiments (e.g. PCR, western blot, and luciferase reporter assay); functional context datasets from MSigDB; experimentally supported miRNA targets from TarBase, miRecord, miR2Disease, and miRTarBase, and miRNA binding sites from miRanda, for each sponge. | 599 miRNA-sponge interactions and 463 ceRNA interactions across 11 species, including endogenous and exogenous sponges, such as pseudogenes, lncRNAs, and circRNAs. | Information on experimentally verified miRNA-sponge and ceRNA interactions, including corresponding literature and graphical visualization of functional annotations, if identified; access to DAVID API tool for users to carry out multi-context functional analyses. | http://bio-bigdata.hrbmu.edu.cn/miRSponge/ | [120] | |
| Co-LncRNA | RNA-seq datasets from GEO and TCGA; LncRNA and protein-coding gene annotation from GENCODE. | Exclusively human transcripts (protein-coding transcripts and lncRNAs) across 28 tissue types/cell lines. | Detection of co-expressed lncRNAs and protein-coding genes under user-selected biological conditions and stringency criteria; graphical visualization of the relevant lncRNA-PCG co-expression networks and the pathways that are modulated by lncRNAs; analysis of lncRNA and protein-coding gene expression profiles uploaded by users. | http://bio-bigdata.hrbmu.edu.cn/Co-LncRNA/ | [121] | |
| Lnc2Meth | Relevant published literature from PubMed; Illumina Infinium Human Methylation 450k BeadChip array datasets from GEO and TCGA; WGBS datasets from ENCODE and TCGA; annotation data from GENCODE. | 471 associations between human lncRNAs and DNA methylation status, as well as 301 differential methylation profiles for lncRNAs and PCGs across 72 types of human diseases. | Annotation of experimentally verified lncRNA-methylation associations; identification and visualization of predicted differential methylation patterns of lncRNAs and PCGs from internal datasets or user-supplied external datasets online or with local computers. | http://bio-bigdata.hrbmu.edu.cn/Lnc2Meth/ | [122] | |
| **lncRNA-disease associations** | | | | | |  |
| LncRNADisease 2.0 | Relevant published literature from PubMed; predicted lncRNA/circRNA-disease associations from LRLSLDA, LDAP, RWRlncD and LncDisease; miRNA annotation data from miRBase; predicted miRNA targets from PITA, miRanda and RNAhybrid. | 19,166 lncRNAs and 823 circRNAs associated with 529 diseases across four species, including humans. | Detailed annotation of the lncRNA-disease associations and assignment of a confidence score to each association based on the evidence level; network viewing of regulatory relationships between disease-related lncRNAs and their mRNA and miRNA targets. | http://www.rnanut.net/lncrnadisease/ | [123,124] | |
| Lnc2Cancer v3.0 | Published literature curated from PubMed containing associations validated by strong experimental evidence (RNAi, in vitro knockdown, western blot, qRT-PCR, luciferase reporter assays, and high-quality high-throughput methods); Annotation data from GENCODE. | 2,775 lncRNAs and 743 circRNAs associated with 226 human cancer subtypes. | Annotation of the lncRNA-cancer associations and assignment of a confidence score to each association based on the number of supporting papers and the sample type in experiments;  classification of the lncRNAs into searchable categories according to their potential role as biomarkers, biological functions, and their regulatory mechanisms in cancer. | http://www.bio-bigdata.net/lnc2cancer/ | [125,126] | |
| MNDR v2.0 | Relevant published literature from PubMed; ncRNA-disease associations from previous version of MNDR, dbDEMC, HMDD, Lnc2Cancer, LincSNP, LncRNADisease, miR2Disease, miRCancer, NSDNA, and PhenomiR; predicted ncRNA-disease associations from LDAP, LncDisease, miRPD, and PBMDA. | Associations between ncRNAs (lncRNAs, miRNAs, piRNAs, and snoRNAs) and diseases across six mammals, including 8,824 experimental and 153,508 predicted entries for humans. | Detailed annotation of the ncRNA-disease associations and assignment of a confidence score to each association based on the evidence level; disease terms based on Disease Ontology and MeSH vocabularies. | http://www.rna-society.org/mndr/ | [127] | |
| ncRPheno | ncRNA-disease association from miR2Disease, miREnvironment, LncRNADisease, DLREFD, circRNADisease, HMDD, SM2miR, Lnc2Cancer, NSDNA, lncRNASNP2, miRNASNP, GWAS Catalog, NPInter, miRSponge, and LncReg; Annotation data from Ensembl, RNAcentral, miRBase, circBase, piRNABank, snoRNA-LBME-db, EFO, ORDO, and ECO. | Associations between 14,494 ncRNAs (lncRNAs, circRNAs, miRNAs, piRNAs, and snoRNAs) and 3,210 disease phenotypes across 11 species, including 416,904 ncRNA-disease associations from humans. | Annotation of the ncRNA-disease associations and assignment of a score to each association based on the evidence level and the number of supporting papers; graphical visualization of the relationships between ncRNAs and diseases, including tree word-clouds and node-link diagrams; ncRNA prioritization application to detect and prioritize experimentally validated ncRNAs to a corresponding disease. | http://lilab2.sysu.edu.cn/ncrpheno | [128] | |
| Lnc2Catlas | SNPs from dbSNP and cancer-related SNPs from GWAS and ClinVar; cancer-related proteins from MalaCards and DisGeNET; and genes from HPA; expression profiles from TCGA; experimentally supported lncRNA-cancer associations from PubMed and several other databases, including lncRNAdb, LNCipedia, NONCODE, LncRNADisease, and Lnc2Cancer; annotation data from GENCODE. | 27,670 lncRNA transcripts across 33 human cancer types. | Information on each cancer-associated lncRNA, including basic annotation data and the results obtained using scoring methods to assess secondary structure disruption by cancer-associated SNPs, interaction with cancer-related proteins, and expression similarity with genes with key roles in cancer; graphical visualization of co-expression networks and lncRNA expression levels across different cancer types. | http://lnc2catlas.bioinfotech.org/ | [129] | |
| CSCD | RNA-Seq datasets from ENCODE; predicted circRNA back-splice junction sites from CIRI2, find_circ, circRNA finder and Circexplorer; predicted MREs and circRNA ORFs from TargetScan and ORF Finder, respectively; CLIP-seq data from starBase; potential alternative splicing events from rMATS for all RNA-seq samples. | 272,152 human cancer-specific circRNAs across 19 cancers types. | Information on cancer-specific circRNAs as well as normal and common circRNAs collected exclusively from cell line samples; graphical visualization of the circRNAs, including their potential ORFs, MREs, protein binding sites and the corresponding linear parent genes. | http://gb.whu.edu.cn/CSCD | [130] | |
| Circ2Disease | Published literature curated from PubMed containing circRNAs and circRNA-associated miRNAs validated by high confidence experiments (evidence supported only by high-throughput data not included); annotation data from circBase and Human circRNA microarray; predicted MREs from TargetScan and miRanda; miRNA-associated diseases from HMDD, dbDEMC, and OncomiRDB. miRNA targets from miRecords and miRTarBase; Potential circRNA interacting RBPs from CircInteractome. | 273 associations between 237 circRNAs and 54 human diseases, including miRNAs putatively sponged by these circRNAs and their validated targets. | Detailed annotation of circRNAs and circRNA-disease associations; network viewing of the circRNAs and their up- and downstream genes as well as their associated diseases. | http://bioinformatics.zju.edu.cn/Circ2Disease/index.html | [131] | |
| CircR2Disease | Relevant published literature from PubMed. | 725 experimentally- supported associations between 661 circRNAs and 100 diseases across humans, rats and mice. | Summarized information on circRNA-disease associations, including external links to the literature referenced in PubMed, the circRNA and the disease annotations in circBase and MalaCards, respectively. | http://bioinfo.snnu.edu.cn/CircR2Disease/ | [132,133] | |
| MiOncoCirc | Exome capture RNA-seq data from previously published studies using clinical samples, cell line panels and pooled normal tissues. | CircRNAs from primary tumors, metastases, and very rare tumors, comprising 40 human cancer types. | Information on circRNAs and read-through circRNAs, including abundance of the circRNAs and the corresponding parent genes across different cancer types. | https://mioncocirc.github.io/ | [134] | |

AGO-CLIP-seq: Argonaute CLIP-seq; ceRNA: competing endogenous RNA; ChIRP-seq: Chromatin Isolation by RNA Purification-seq; circRNA: circular RNA; CLIP-seq: High-throughput sequencing of immunoprecipitated RNAs after crosslinking; COME: coding potential calculator based on multiple features; COSMIC: Catalogue of Somatic Mutations in Cancer; CPC: The Coding Potential Calculator; CSCD: Cancer-specific circRNA Database; dbDEMC: database of differentially expressed miRNAs in human cancer; DLREFD: The Disease Related LncRNA-Environmental Factors Interaction Database; ECO: Evidence & Conclusion Ontology; EFO: Experimental Factor Ontology; ENCODE: The Encyclopedia of DNA Elements; ENCORI: The Encyclopedia of RNA Interactomes; FANTOM CAT: Functional Annotation of the Mammalian Genome - CAGE associated transcriptome; GEO: Gene Expression Omnibus; GO: Gene Ontology; GTEx: The Genotype-Tissue Expression project; GWAS: Genome-wide Association Studies; H-InvDB: H-Invitational Database; HMDD: The Human microRNA Disease Database; HPA: Human Protein Atlas; KEGG: Kyoto Encyclopedia of Genes and Genomes; LDAP: LncRNA-Disease Association Prediction; lncRNA: long noncoding RNA; LRLSLDA: Laplacian Regularized Least Squares for LncRNA-Disease Association; MeSH: Medical Subject Headings; miRNA: microRNA; MNDR: Mammal ncRNA-Disease Repository; MREs: miRNA response elements; MSigDB: Molecular Signatures Database; NCBI: National Center for Biotechnology Information; ncRNA: noncoding RNA; NSDNA: The Nervous System Disease ncRNAome Atlas; ORDO: Orphanet Rare Disease Ontology; ORF: Open Reading Frame; PANTHER: Protein Analysis Through Evolutionary Relationships; PBMDA: Path-Based miRNA-Disease Association; PCGs: protein-coding genes; piRNA: piwi-interacting RNA; qRT-PCR: quantitative reverse transcription polymerase chain reaction; RBPs: RNA-binding proteins; RNAi: RNA interference; snoRNA: small nucleolar RNA; SNP: single nucleotide polymorphism; TCGA: The Cancer Genome Atlas; WGBS: Whole Genome Bisulfite Sequencing.
